# Molecular prevalence of *Theileria* infections in cattle in Yanbian, north-eastern China

**DOI:** 10.1051/parasite/2020017

**Published:** 2020-03-30

**Authors:** Lijun Jia, Shaowei Zhao, Suzhu Xie, Hang Li, Hao Wang, Shuang Zhang

**Affiliations:** Laboratory of Veterinary Microbiology, Department of Veterinary Medicine, Agriculture College of Yanbian University No. 977 Park Road 133000 Yanji PR China

**Keywords:** *Theileria orientalis*, *Theileria sinensis*, Yanbian, Epidemiology

## Abstract

Bovine *Theileria* are tick-borne protozoan parasites that invade bovine erythrocytes and lymphocytes. Three main bovine *Theileria* species have been identified in China: *T. orientalis*, *T. sinensis*, and *T. annulata*. To examine the prevalence of bovine theileriosis in Yanbian, a total of 584 bovine blood samples were collected from five localities from 2017 to 2019 and analyzed by PCR. Six pairs of oligonucleotide primers directed against the 18S rRNA gene of *Theileria* spp., Tams-1 gene of *T. annulata*, MPSP gene of *T. orientalis*, and *T. sinensis*, were used to detect these parasites. A sequence analysis of the amplified genes confirmed that the *Theileria* species were *T. orientalis* and *T. sinensis*, without *T. annulata*. The overall prevalence of *Theileria* in cattle was 42.81% (250/584). Out of the 584 samples, 159 (27.23%) and 157 (26.88%) were positive for *T. sinensis* and *T. orientalis*, respectively, and the mixed infection rate was 11.30% (66/584). The total prevalence of bovine *Theileria* species in Helong, Hunchun, Longjing, Yanji, and Dunhua was 66.28%, 49.68%, 23.81%, 28.15%, and 0%, respectively. These results provide epidemiological data for the prevention and control of bovine *Theileria* species in Yanbian, China.

## Abstract (fr)

Les *Theileria* bovins sont des parasites protozoaires transmis par les tiques qui envahissent les érythrocytes et les lymphocytes des bovins. Trois espèces principales de *Theileria* de bovins ont été identifiées en Chine, *T. orientalis*, *T. sinensis* et *T. annulata*. Pour examiner la prévalence de la theilériose bovine à Yanbian, un total de 584 échantillons de sang bovin ont été collectés dans cinq localités de 2017 à 2019 et analysés par PCR. Six paires d’amorces oligonucléotidiques dirigées contre le gène d’ARNr 18S de *Theileria* spp., le gène Tams-1 de *T. annulata* et le gène MPSP de *T. orientalis* et *T. sinensis*, ont été utilisées pour détecter ces parasites. Une analyse de séquence des gènes amplifiés a confirmé que les espèces de *Theileria* étaient *T. orientalis* et *T. sinensis*, sans *T. annulata*. La prévalence globale des *Theileria* chez les bovins était de 42,81 % (250/584). Sur les 584 échantillons, 159 (27,23 %) et 157 (26,88 %) étaient positifs pour *T. sinensis* et *T. orientalis*, respectivement, et le taux d’infection mixte était de 11,30 % (66/584). La prévalence totale des espèces bovines de *Theileria* à Helong, Hunchun, Longjing, Yanji et Dunhua était respectivement de 66,28 %, 49,68 %, 23,81 %, 28,15 % et 0 %. Ces résultats fournissent des données épidémiologiques pour la prévention et le contrôle des espèces de *Theileria* de bovins à Yanbian, en Chine.

## Introduction

Bovine theileriosis, which primarily causes fever, anaemia, jaundice, and superficial lymph node enlargement in infected animals, is a tick-borne haemoprotozoan disease caused by parasites of the genus *Theileria,* which invades bovine erythrocytes and leukocytes [12]. The prevalence and active regions of vector ticks are critical components of bovine *Theileria* species epidemiology. The disease, which is difficult to completely eliminate, is associated with obvious regional and seasonal epidemics. Severe infections of cattle result in death, which causes considerable economic losses and potential threats to the cattle industry [24]. It has currently been established that the causative agents of bovine theileriosis include *Theileria parva*, *Theileria annulata*, *Theileria mutans*, and *Theileria velifera* [15]. *Theileria parva* and *Theileria annulata* cause a higher mortality rate in cattle, and represent two of the most virulent species compared with the other reported *Theileria* species. *Theileria annulata* is widely distributed throughout Europe, the Middle East, Russia, China, and Africa [22], whereas *Theileria parva*, termed East Coast fever, is primarily distributed in Africa [1]. In these regions where the cattle industry has developed, the economic losses to the industry related to *T. annulata* are higher than the losses related to *Theileria* species.

In China, the reported bovine *Theileria* species mainly include *T. orientalis*, *T. sinensis*, and *T. annulata* [26], which are widely distributed and constitute a considerable threat. *Theileria orientalis* is a haemoprotozoan parasite that infects cattle and buffalo, and is typically transmitted by *Haemaphysalis* ticks [10]. Previous studies have referred to this parasite as *T. sergenti*, *T. buffeli*, or a mixture of *T. orientalis*, *T. buffeli*, and *T. sergenti*; however, *T. sergenti* has now been replaced by *T. orientalis*. *Theileria sinensis* exhibits relatively weak pathogenicity, and is primarily distributed throughout Asia (e.g., Japan, China, and the Korean Peninsula) [7]. *Theileria sinensis* in China was identified in cattle and yak distributed in Gansu and the Qinghai-Tibet Plateau; however, the pathogenicity of the parasite requires further research. *Theileria sinensis* was first isolated in Gansu, China by Bai et al. from cattle naturally infected with parasites. To determine the taxonomic status of the indeterminate *Theileria* species, this parasite was compared with other bovine *Theileria* species by Chinese scholars using morphological comparisons, inoculation tests, and host-specific tests, and was finally termed *Theileria sinensis* [3]. *Theileria annulata* is propagated by deadly tick species of the genus *Hyalomma*, and is widely distributed throughout North Africa, Southern Europe, India, the Middle East, and Central Asia [25]. The life cycle of *T. annulata* is highly complex, and involves two stages: (1) haploid vegetative propagation in cattle; and (2) diploid sexual reproduction in ticks [5].

Yanbian is located in north-eastern China in the Golden Triangle of north-eastern Asia, and is bordered by North Korea to the south, and Russia to the east. In this study, a total of 584 blood samples of cattle were obtained and tested for the molecular detection of bovine *Theileria* and species identification.

## Materials and methods

### Ethics

Farm owners were contacted and permissions were obtained to have their animals involved. All experimental procedures in animals were conducted following the Ethical Principles in Animal Research issued by Yanbian University.

### Sample collection and DNA extraction

In total, 584 cattle blood samples were obtained from the following five counties in Yanbian between 2017 and 2019: Helong (172), Hunchun (157), Longjing (84), Yanji (135), and Dunhua (36). Approximately 10 mL of blood was aseptically collected from the jugular vein of each animal using vacuum tubes, and stored at −20 °C until DNA extraction. The DNA was extracted from the whole blood using a blood extraction kit (OMEGA) and then stored at −20 °C until future use.

### Primer design and synthesis

According to the sequences for the *Theileria* spp. 18S rRNA [4], *T. annulata* Tams-1 [14], *T. orientalis* MPSP [16], and *T. sinensis* MPSP [13] genes, six pairs of primers were synthesized by Shanghai Yingjun Biotechnology Co., Ltd. The primer sequences are listed in Table 1.

**Table 1:** Primer sequences

Pathogen	Target gene	Assay	Oligonucleotide sequences (5′ → 3′)	Product size (bp)	Annealing temperature (°C)	Reference
*Theileria* spp	18S rRNA	PCR	GAAACGGCTACCACATCT	778	55	Cao et al. [4]
			AGTTTCCCCGTGTTGAGT			
		nPCR	TTAAACCTCTTCCAGAGT	581	55	
			TCAGCCTTGCGACCATAC			
*T. annulata*	Tams-1	PCR	GTAACCTTTAAAAACGT	721	55	Martin-Sanchez et al. [14]
			GTTACGAACATGGGTTT			
		nPCR	CACCTCAAAACATACCCC	453	60	
			TGACCCACTTATCGTCC			
*T. orientalis*	MPSP	PCR	CTTTGCCTAGGATACTTCCT	776	58	Ota et al. [16]
			ACGGCAAGTGGTGAGAACT			
*T. sinensis*	MPSP	PCR	CACTGCTATGTTGTCCAAGAGATATT	887	56	Liu et al. [13]
			AATGCGCCTAAAGATAGTAGAAAAC			

### Bovine *Theileria* DNA amplification

Genomic DNA was used as a template for conventional PCR and nested PCR (nPCR) amplification (Table 1). Genomic DNA isolated from cattle infected with *T. orientalis* and distilled water were used as positive and negative controls, respectively. The PCR reaction was conducted in a 20-μL reaction mixture comprising 1.0 μL of each primer (10 pmol), 4.0 μL template DNA (50 ng/μL), 2.0 μL dNTP Mix (Baosheng Dalian Bioengineering Co. Ltd), 2.0 μL of 10 × Ex Taq buffer, 1 μL Ex Taq (Baosheng Dalian Bioengineering Co. Ltd), and 9 μL distilled water. The amplification conditions consisted of an initial denaturation step at 95 °C for 5 min, followed by 30 cycles of denaturation at 94 °C for 45 s, an annealing step at the temperature set for each primer for 1 min, an extension step at 72 °C for 1 min, and a final extension at 72 °C for 7 min. The annealing temperatures are presented in Table 1.

### Sequencing and phylogenetic analysis

Amplicons from positive PCR products were cloned into the PMD 18T-Simple Vector (Baoshengwu, Dalian, China) and transformed into competent DH5α cells. Plasmid DNA was extracted using a plasmid extraction kit (OMEGA), and further identified by PCR and double enzyme digestion by restriction endonucleases SaI I and BamH I (Baoshengwu, Dalian, China). The precisely identified products were sent to Shanghai Yingjun Biotechnology Company for sequencing.

The sequences correctly obtained from the present study were subjected to a BLAST analysis using the BLASTn programme in the NCBI GenBank. Multiple alignments and phylogenetic analyses of the obtained sequences of the 18S rRNA and MPSP genes of bovine *Theileria* were performed using Clustal W [20] (BioEdit version 7.0.9) and the maximum likelihood (ML) (MEGA version 7 software) and Bayesian (MrBayes version 3.2) methods [19]. The substitution model Tamura-3-parameter was used for maximum likelihood (ML) analyses. The search for the ML tree and bootstrap resampling with 1000 replications were performed using MEGA. In the Bayesian analysis, the GTR + G + I model (*n* = 6, rates = invgamma) was selected to perform for 10^6^ generations with sampling every 10^3^ generations and the initial 25% of the sampled trees were discarded as burn-in. The GenBank accession numbers of the relative species used in this study are shown in Figure 2.

## Results

### Molecular prevalence of bovine *Theileria*

**Figure 1:**
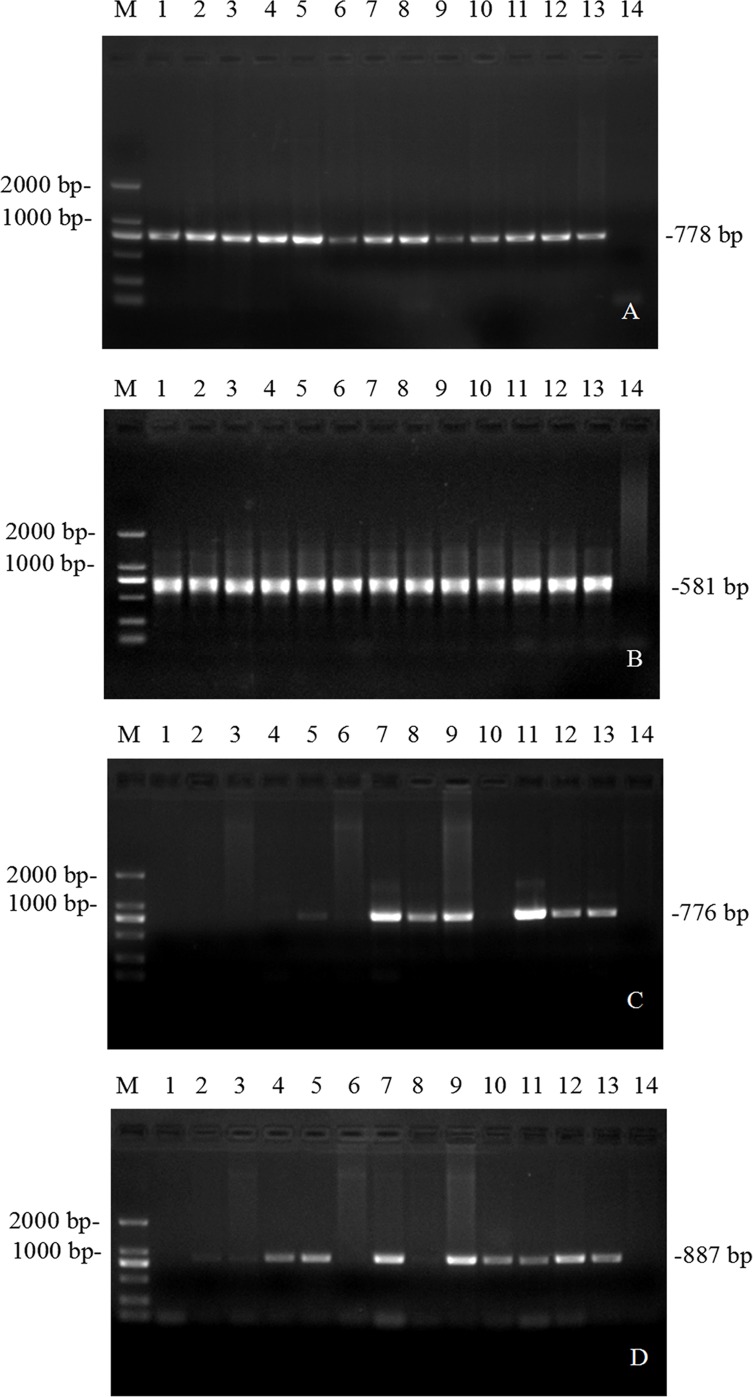
(A) and (B) *Theileria* spp. nested PCR, M: DL 2 000 DNA Marker, lain1: Positive control, lain2-13: Sample products, lain14: Negative control. (C) and (D) *T. orientalis* PCR and *T. sinensis* PCR, M: DL 2000 DNA Marker, lain1-13: PCR products of the target gene, lain14: Negative control.

Primers designed from the *Theileria* spp., *T. annulata*, *T. sinensis*, and *T. orientalis* genes were used for detection of DNA in the 584 bovine blood samples by PCR. The PCR amplification results show that *Theileria* spp. PCR amplified two fragments consisting of 778 bp (P1, P2) and 581 bp (P3, P4). *Theileria sinensis* PCR and *Theileria orientalis* PCR amplified fragments consisting of 887 bp (P5, P6) and 776 bp (P7, P8), respectively. However, all of the blood samples tested negative for *T. annulata*. The PCR amplification results for *Theileri*a spp., *T. sinensis*, and *T. orientalis* are shown in Figure 1A–D.

Among the 584 sampled animals, the prevalence of *T. sinensis* and *T. orientalis* infection was 27.23% (159/584) and 26.88% (157/584), respectively. Additionally *T. annulata* was not found in our study. The mixed infection rate for *T. sinensis* and *T. orientalis* was 11.30% (66/584) in this study (Table 2).

**Table 2:** The single and mixed infection rates of *Theileria* spp. in cattle in Jilin, China.

	Helong (*n* = 172)				Hunchun (*n* = 157)		Longjing (*n* = 84)		Yanji (*n* = 135)		Dunhua (*n* = 36)		Total (*n* = 584)	
	No. of positive		95% CI		No. of positive	95% CI	No. of positive	95% CI	No. of positive	95% CI	No. of positive	95% CI	No. of positive	95% CI
Single infection														
*T. sinensis*	42		24.42 (18.60–31.35)	31		19.75 (14.27–26.66)	4	4.76 (1.87–11.61)	16	11.85 (7.43–18.39)	0	0 (0–9.64)	93	15.92 (13.18-19.11)
*T. orientalis*	44		25.58 (19.64–32.59)	25		15.92 (11.02–22.45)	8	9.52 (4.91–17.68)	14	10.37 (6.28–16.65)	0	0 (0–9.64)	91	15.58 (12.87–18.75)
Mixed infection														
*T. sinensis* + *T. orientalis*		28	16.28 (11.51–22.52)	22		14.01 (9.44–20.30)	8	9.52 (4.91–17.68)	8	5.93 (3.03–11.26)	0	0 (0–9.64)	66	11.30 (8.98–14.13)
Total positive		114	66.28 (58.93–72.92)	78		49.68 (41.96–57.42)	20	23.81 (15.98–33.93)	38	28.15 (21.25–36.26)	0	0 (0–9.64)	250	42.81 (38.86–46.86)
Negative samples		58	33.72 (27.08–41.07)	79		50.32 (42.58–58.04)	64	76.19 (66.07–84.02)	97	71.85 (63.74–78.75)	36	100 (90.36-100)	334	57.19 (53.14–61.14)

### Target gene cloning and identification

The recombined clone plasmids PMD18-T-*Theileria* spp., PMD18-T-*T. sinensis*, and PMD18-T-*T. orientalis* were constructed with positive PCR products and clone vectors. Three gene fragments comprised of 581 bp, 887 bp, and 776 bp were obtained by PCR. Positive clones of each target gene were digested with BamH I and SaI I, and 581 bp, 2692 bp, 887 bp, 2692 bp, 776 bp, and 2692 bp fragments were obtained.

### Comparative analysis

The 18S rRNA gene of *Theileria* spp., *T. orientalis* MPSP gene, and *T. sinensis* MPSP gene were identified in this study. Nucleotide sequence identity data demonstrated that the sequence of the 18S rRNA (MN628025) gene shares 100% sequence identity with China (KX115427.1). The MPSP gene (887 bp) of *T. sinensis* isolated in this study was 100% identical to a previously published sequence from Jilin (KX375400.1). Similarly, the *T. orientalis* gene obtained in our study had 99.6% nucleotide homology with Jilin 2 (KY392962.1), and three nucleotide mutations were found. Representative *T. orientalis* and *T. sinensis* MPSP sequences for different strains were registered in the GenBank database under accession numbers: MN630024 – MN630031.

### Phylogenetic analysis

**Figure 2:**
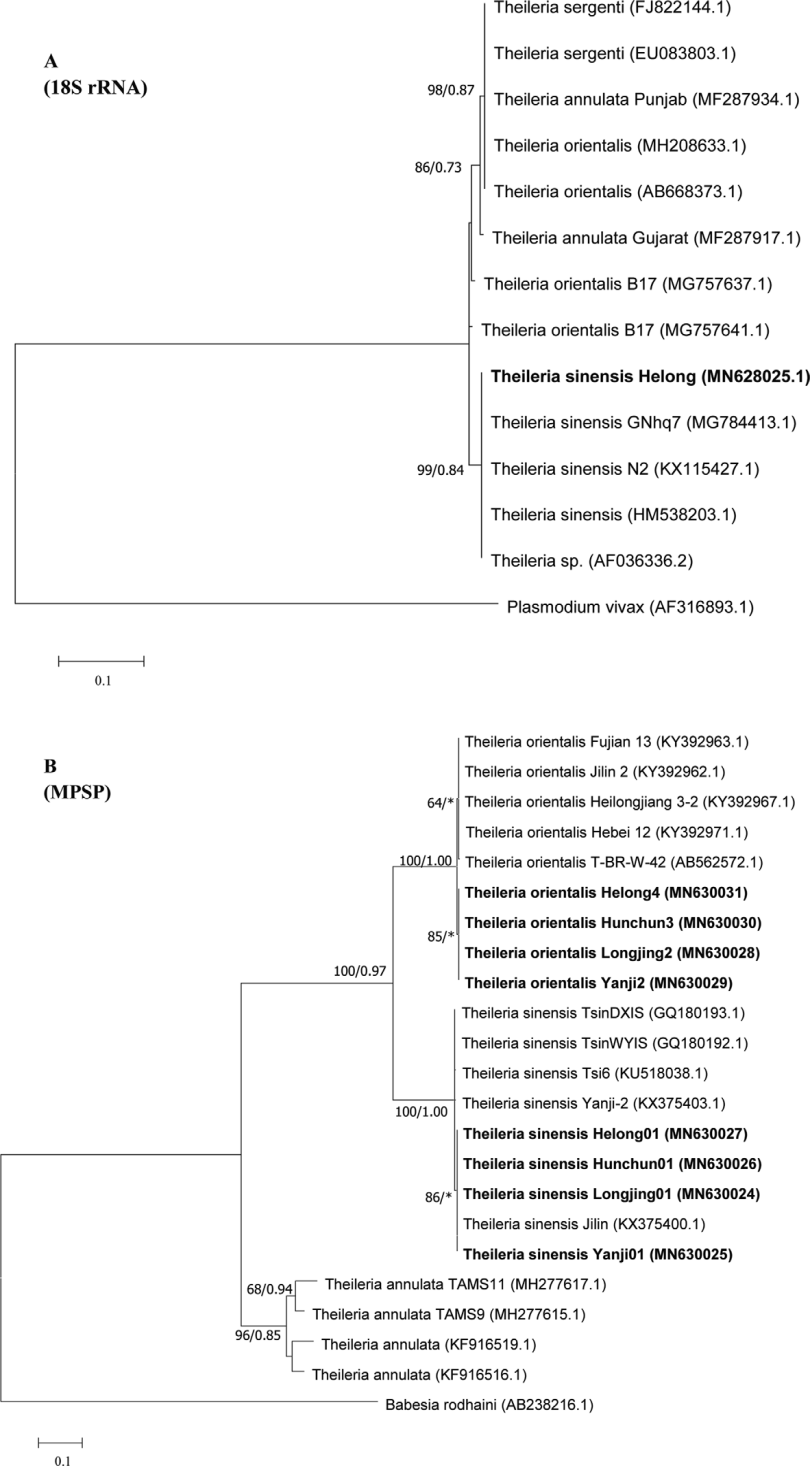
Phylogenetic trees based on the (A) 18SrRNA and (B) MPSP sequences of bovine *Theileria.* The ML tree was derived from a Tamura 3-parameter model using MEGA7, and Bayesian Inference by Mrbayes3.2 with the GTR + G + I model. ML bootstrap and BI posterior probabilities values are shown at the nodes in the order ML/BI. Bootstrap values <50 are not reported. Posterior probabilities <0.7 are not reported. The newly generated sequences in the present study are shown in bold.

Two phylogenetic trees of bovine *Theileria* were constructed from the 18S rRNA and MPSP gene sequences of our amplicons and those available in GenBank. ML and BI analyses generated phylogenetic trees with similar topologies. A single tree topology was presented with support values (ML/BI). The sequence of the 18S rRNA gene obtained from our study (MN628025) was 100% identical to that of Guo (MG784413.1) isolated from *Haemaphysalis qinghaiensis*. The 18S rRNA sequence of *T. sinensis* described here formed a well-supported clade with all the studied *T. sinensis*, while the other *Theileria* species belonged to different clades such as *T. sergenti* (Fig. 2A). Concerning the MPSP gene, the phylogenetic analysis showed evidence of three main clades, one consisting of *T. sinensis* and the others *T. orientalis* and *T. annulata* (Fig. 2B). The phylogenetic analysis indicated that the *T. orientalis* MPSP gene from this study formed one cluster with the isolates from Fujian (KY392963.1), Heilongjiang (KY392967.1), and Thailand (AB562572.1). The isolated *T. sinensis* formed one clade with Jilin (KX375400.1). In addition, the MPSP genes of the *T. orientalis* isolated in this study were classified near the cluster of *T. sinensis* rather than that of *T. annulata.*

## Discussion

*Theileria orientalis* infection occurs most frequently from June to July and September to October, and is the type of bovine theileriosis that spreads most widely throughout China, including in Heilongjiang, Jilin, Hebei, Guangxi, Fujian, Gansu, and Qinghai [6, 11]. *Theileria sinensis* was first isolated from Lintao, China, and represents a new species that primarily infects cattle and yak, and is the most common in the middle region of Gansu Province. However, the prevalence of *T. sinensis* in other regions remains poorly understood [2]. Moreover, *T. annulata* is widely distributed throughout the world, including in Central Asia, North America, and South Africa. The distribution of this parasite in China mainly includes the desert and semi-desert grasslands in the northwest, as well as North China, Greater Khingan, and the Changbai Mountains in the northeast region. Among these regions, Xinjiang has the highest incidence of *T. annulata*. Bovine theileriosis is closely related to the activity of ticks and is characterised by seasonality and regionality, making it difficult to completely eliminate. Severe disease caused by bovine *Theileria* in cattle often leads to death, which causes massive economic losses and represents a potential threat for the cattle industry. Yanbian has a long border and is rich in vegetation resources in the eastern mountainous area. Pasture, forest, and barn environments are particularly suitable for tick breeding and reproduction. Therefore, there are various types of ticks in Yanbian including *Haemaphysalis longicornis*, *Dermacentor silvarum*, and *Ixodes persulcatus*, which are the major vectors responsible for transmitting bovine *Theileria* species.

Our results in Yanbian were that Helong had the highest prevalence of bovine *Theileria* species, followed by Hunchun, Yanji, and Longjing, whereas Dunhua had 0% prevalence. The difference in the positive rate of bovine *Theileria* species among these regions may be directly related to the various feeding conditions of cattle. This is because cattle in extensive grazing conditions have more opportunities to be exposed to vector ticks, which leads to an increased percentage of infection with bovine *Theileria*. In the present study, blood samples collected from Dunhua were from cattle in captivity, whereas the samples from the other four regions were collected from semi-grazing cattle, which may explain the low prevalence of bovine *Theileria* species in Dunhua. Furthermore, the Helong region is located in the Eastern Foot of the Changbai Mountains, where there may be an increase in the species and quantity of the ticks. Therefore, the higher prevalence detected in Helong may be closely related to the geographical location [17].

Molecular taxonomic data for bovine *Theileria* have been relatively absent in China. Since the *Theileria* spp. gene is a highly effective molecular marker sequence, the genotypes and phylogenetic analysis of these parasites are usually studied by researchers in China and abroad utilizing this gene [8]. The rates of infection found in our study were much higher than those for *T. sinensis* (17.5%), *T. orientalis* (10.9%), and co-infection with both parasites (8.8%) detected by Jia et al. [9]. In addition, *T. sinensis* was found to be primarily distributed in the high altitude regions of China, and is rarely found in the lower elevations of northern China [21]. In a previous study, *T. annulata* infection was reported in neighbouring countries and in southern China [23]; however, no infection was found in this study, which may be related to the distribution of tick species in the sampling areas. Due to the limited sample size and geographical sampling location, *T. annulata* infections were not found in our research, which does not mean that no *T. annulata* is present in Yanbian. Therefore, further investigation is required to clarify this finding.

Currently, considerable economic losses to the cattle industry have been caused by the widespread distribution of bovine *Theileria* in China, which also imposes significant constraints on the export of agricultural products [18]. To further understand the epidemic characteristics and regional distribution of bovine *Theileria*, the specimens collected from five counties in Yanbian were analysed by molecular detection. Moreover, positive specimens were assessed by sequencing and phylogenetic analysis utilising the *Theileria* spp. 18S rRNA, *T. orientalis* MPSP, and *T. sinensis* MPSP genes, which aimed to elucidate the epidemic strains of bovine *Theileria* species in Yanbian. These results show that *T. orientalis* and *T. sinensis* are two major epidemic strains in this region. The findings of this study provide scientific evidence for the prevalence and geographical distribution of bovine *Theileria* in the north-eastern frontier of China. This information is useful for the early prevention and control of bovine theileriosis, which can be used to reduce the harm caused by the disease in this region.

## Conclusions

*Theileria sinensis* (27.23%) and *T. orientalis* (26.88%) were found in this study. The findings of our study indicated that Yanbian was an epidemic area of bovine theileriosis. Additionally, feeding conditions and the geographical position of farms are two potential risk factors for the prevalence of bovine theileriosis, which is supported by this paper.
